# Reactions to Media Violence: It’s in the Brain of the Beholder

**DOI:** 10.1371/journal.pone.0107260

**Published:** 2014-09-10

**Authors:** Nelly Alia-Klein, Gene-Jack Wang, Rebecca N. Preston-Campbell, Scott J. Moeller, Muhammad A. Parvaz, Wei Zhu, Millard C. Jayne, Chris Wong, Dardo Tomasi, Rita Z. Goldstein, Joanna S. Fowler, Nora D. Volkow

**Affiliations:** 1 Department of Psychiatry, Friedman Brain Institute, Icahn School of Medicine at Mount Sinai, New York, New York, United States of America; 2 Department of Neuroscience, Friedman Brain Institute, Icahn School of Medicine at Mount Sinai, New York, New York, United States of America; 3 Laboratory of Neuroimaging, National Institute on Alcohol Abuse and Alcoholism, Bethesda, Maryland, United States of America; 4 Applied Mathematics and Statistics, SUNY, Stony Brook, New York, United States of America; 5 Medical Department, Brookhaven National Laboratory, Upton, New York, United States of America; Glasgow University, United Kingdom

## Abstract

Media portraying violence is part of daily exposures. The extent to which violent media exposure impacts brain and behavior has been debated. Yet there is not enough experimental data to inform this debate. We hypothesize that reaction to violent media is critically dependent on personality/trait differences between viewers, where those with the propensity for physical assault will respond to the media differently than controls. The source of the variability, we further hypothesize, is reflected in autonomic response and brain functioning that differentiate those with aggression tendencies from others. To test this hypothesis we pre-selected a group of aggressive individuals and non-aggressive controls from the normal healthy population; we documented brain, blood-pressure, and behavioral responses during resting baseline and while the groups were watching media violence and emotional media that did not portray violence. Positron Emission Tomography was used with [^18^F]fluoro-deoxyglucose (FDG) to image brain metabolic activity, a marker of brain function, during rest and during film viewing while blood-pressure and mood ratings were intermittently collected. Results pointed to robust resting baseline differences between groups. Aggressive individuals had lower relative glucose metabolism in the medial orbitofrontal cortex correlating with poor self-control and greater glucose metabolism in other regions of the default-mode network (DMN) where precuneus correlated with negative emotionality. These brain results were similar while watching the violent media, during which aggressive viewers reported being more *Inspired* and *Determined* and less *Upset* and *Nervous*, and also showed a progressive decline in systolic blood-pressure compared to controls. Furthermore, the blood-pressure and brain activation in orbitofrontal cortex and precuneus were differentially coupled between the groups. These results demonstrate that individual differences in trait aggression strongly couple with brain, behavioral, and autonomic reactivity to media violence which should factor into debates about the impact of media violence on the public.

## Introduction

While visual media is replete with images of violence, only a small minority in the population engages in real-life violent behavior. Critically, whether a person will act violently depends on individual trait variations which play a prominent role in how visual media is experienced and processed [1]. Therefore, understanding the neurobiological underpinnings of those with aggressive personality traits above the documented norms, is an important prerequisite to the ongoing debate about media impact on behavior [2]. Enduring trait aggression reflects self-report of retaliatory motivation, with high face validity, where individuals endorse questions regarding the degree of their readiness to hurt others. It is emerging in the literature that aggressive individuals differ from non-aggressive individuals in their baseline, trait-like, neurobiological architecture [3], suggesting involvement of the brain’s default mode network (DMN) [4], [5]. The DMN forms a distributed circuit of connected brain systems that shows high and coherent metabolic activity or blood flow during awake yet passive resting states which may represent internal and self-referential processing [4]–[7]. The DMN includes regions typically spanning the posterior cingulate cortex (PCC) and precuneus, lateral inferior parietal gyrus (IPG), medial temporal gyrus (MTG), and ventromedial prefrontal cortex, including the orbitofrontal cortex (OFC) [8]. We hypothesize that at resting baseline, individuals with high trait aggression will exhibit different brain metabolism patterns in the DMN including its ventromedial prefrontal regions, revealing fundamentally different internal preoccupations than those with normative trait aggression.

Stimuli with violent themes can prime, or perhaps facilitate existing trait tendencies [1], [9]. The General Aggression Model (GAM) [10] outlines the processes by which exposure to violence can cause aggressive behavior through the interplay of enduring traits that drive internal states, coupled with congruent visual stimuli from the environment (e.g., violent media). Therefore, according to GAM, chronic exposure to violent images in the media reinforces existing aggressive traits, thereby preparing the individual towards future violence [11], [12]. The OFC is specifically involved in elements of aggressive behaviors [13]–[15] through its role in prioritizing emotional cues according to intrinsic salience [16]. Likewise, gray matter deficits in the OFC have been observed in individuals with aggressive and violent behavior [17]. As such, we predict involvement of the OFC since it appears to be specifically involved in response to repeated media violence [18], [19]. Individual differences in brain and behavior during visual media viewing can be further understood in the context of self-reported affective states and autonomic responses (or lack thereof) [20], [21]. For example, self-reported distress and systolic blood pressure changes were observed in response to viewing violent media [1], [21]. Cortical representations of emotion-dependent autonomic response (e.g., blood pressure) have been shown in the OFC, anterior cingulate, and insula in response to viewing violent media in healthy controls [22].

To test our hypotheses regarding baseline and media viewing differences as a function of trait aggression, we recruited a group of healthy aggressive individuals with a history of assault behavior and a group of non-aggressive healthy controls. Measurements of glucose metabolism with [^18^F]fluoro-deoxyglucose using positron emission tomography (PET) were obtained at three conditions: at resting baseline, during exposure to violent media, and during exposure to emotional, non-violent media. Blood pressure (BP) and behavioral ratings of state affect were collected intermittently during the movie presentations. We expected that aggressive individuals would have a distinct intrinsic brain activity pattern at resting baseline and during passive viewing of the violent media compared to emotional media.

## Methods

### Ethics Statement

This research protocol was approved by the ethical review board of Stony Brook University and conducted accordingly. All participants provided written informed consent prior to participation. Approval number BNL-381.

### Participants

A total of 54 males who responded to advertisement for healthy controls and healthy individuals with history of physical fights, were evaluated for their physical assault tendencies and other inclusion/exclusion criteria. Individuals were initially screened by phone and then seen at Brookhaven National Laboratory by a physician for general exclusion criteria which included current or past psychiatric disorders (e.g., drug abuse or dependence), neurological disease, significant medical illness, current treatment with medication (including over the counter drugs) and head trauma with loss of consciousness >30 minutes. Normal physical examination and laboratory tests were required for entry and pre-scan urine tests ensured the absence of any psychoactive drugs. Individuals were classified as aggressive (Ag) or non-aggressive (Na) depending on their responses on the Physical Aggression subscale of the Buss-Perry Aggression Questionnaire (the physical aggression subscale correlates strongly with peer ratings of aggression demonstrating its concurrent validity) [23]. Of these 54 participants, only individuals who reported physical fights in the last year and scored at or higher than 75^th^ percentile on the Physical Aggression scale (Ag, n = 12) or those who reported they did not engage in physical fights and scored at 50^th^ percentile or below on the Physical Aggression scale (Na, n = 13) were chosen for the study (mean age 25.15) [23]. As planned, the participants differed on Physical Aggression (Ag, mean ± standard error 33.5±1.2; Na, 14.5±1.0, p<.0001). They also differed significantly on the other subscales of the Buss-Perry: Verbal Aggression (Ag, 18.8±1.0; Na, 11.6±1.2, p<.0001), Anger (Ag, 23.7±1.5; Na, 9.6±0.6, p<.0001), Hostility (Ag, 23.1±2.0; Na, 11.8±0.9, p<.0001) and the total score (Ag, 99.5±3.8; Na, 47.5±2.7, p<.0001). The two groups did not differ on age, handedness [24], socio-economic status [25], estimates of verbal and non-verbal intelligence [26], [27], and depression symptoms [28]. Participants were asked about their media habits including the number of hours they watched TV per day on weekdays and on weekends (Table 1). The participants were monetarily compensated for their participation. It is important to note that the staff performing the media exposure, imaging, nursing, and questionnaire completion, were blind to the subject’s assignment as aggressive or non-aggressive.

**Table 1:** Demographics, personality, inhibitory control, and media exposure as a function of trait aggression.

Demographics^a^	Ag	Na	Statistics
Age	24.9±0.8	25.4±0.8	t_23_ = −0.4, P = 0.69
Laterality Quotient	0.86±0.07	0.92±0.02	t_21_ = −0.8, P = 0.42
SES	42.8±3.2	44.7±3.4	t_21_ = −0.3, P = 0.69
WRAT-3	105.1±2.9	110.7±2.5	t_21_ = −1.4, P = 0.16
MATRIX	10.7±0.7	12.5±0.6	t_21_ = −1.8, P = 0.08
BDI	7.0±1.3	4.6±0.90	t_21_ = −1.5, P = 0.15
Personality			
**Negative Emotionality**	**28.1±2.5**	**7.9±2.3**	**t_21_ = 5.8, P = 0.0001**
** Alienation**	**7.1±1.4**	**1.7±0.57**	**t_21_ = 3.7, P = 0.001**
** Aggression**	**13.7±1.0**	**3.6±1.1**	**t_21_ = 6.3, P = 0.0001**
** Stress Reaction**	**7.1±0.95**	**2.5±0.90**	**t_21_ = 3.5, P = 0.002**
Positive Emotionality	51.2±4.1	47.6±2.5	t_21_ = .73, P = 0.463
Well Being	8.3±0.60	8.5±0.62	t_21_ = −.15, P = 0.877
Social Potency	17.6±1.8	11.6±1.5	t_21_ = 2.5, P = 0.021
Social Closeness	13.0±1.9	14.0±1.9	t_21_ = −.38, P = 0.706
Achievement	12.1±1.3	13.4±1.2	t_21_ = −.68, P = 0.501
Inhibitory Control			
Constraint	44.4±3.9	51.1±2.8	t_21_ = −1.40, P = 0.176
** Control**	**14.0±1.3**	**19.0±0.99**	**t_21_ = −2.98, P = 0.007**
Harm Avoidance	14.0±2.4	17.5±1.8	t_21_ = −1.16, P = 0.256
ANT			
Alerting	35.8±19.0	26.5±6.8	t_21_ = .55, P = 0.615
Orienting	26.3±8.2	44.6±8.9	t_21_ = −1.45, P = 0.159
** Conflict**	**188.2±21.1**	**95.5±7.2**	**t_21_ = 4.6, P = 0.002**
Media Exposure (hours of TV viewed per day)			
On weekdays	3.9±1.4	3.5±2.1	t_21_ = .62, P = 0.798
On weekend	5.6±2.6	4.2±2.4	t_21_ = 1.05, P = 0.278
Most time in a given day	10.8±4.8	9.4±1.9	t_21_ = 1.21, P = 0.310

a Means ± Standard Error, SES: socioeconomic status, WRAT-3: estimate of verbal intelligence, MATRIX: estimate of non-verbal intelligence; BDI: Beck Depression Inventory; ANT attention network task.

### Personality and Behavioral Measures

In addition to the Buss-Perry Aggression Questionnaire, the Multidimensional Personality Questionnaire (MPQ) [29], a three-factor structural model of personality was used. As listed in Table 1, the MPQ models three higher order dimensions of personality: *Negative Emotionality* (NEM, or *Neuroticism*) reflecting tendency toward emotional distress, alienation from others and aggressive behavior; *Positive Emotionality* (PEM, or extraversion) reflecting enduring positive affect through interpersonal engagement, and *Constraint* measuring tendencies toward self-control. Several lines of evidence have shown that high levels of NEM as *Neuroticism* are robustly associated with violence and aggression [30]. Similarly, individuals with elevated scores of NEM tend to experience/report more frequent negative emotions such as anger and anxiety, perceive their environment as hostile/unfair, and often exhibit poor coping mechanisms in a stressful situation [31]. The three NEM sub-scales include *Stress Reaction* which is linked to low frustration tolerance; *Aggression* which reflects the tendency to respond with retaliatory response style; and *Alienation* which is the most predictive primary scale of aggressive behavior. We also assessed attention and inhibitory control using a performance based measure, the Attention Network Task (ANT), that captures reaction-time performance on Alerting (response readiness), Orienting (scanning and selection), and Conflict (inhibitory control) in attention [32].

### Imaging Conditions and State Reactivity

**Figure 1:**
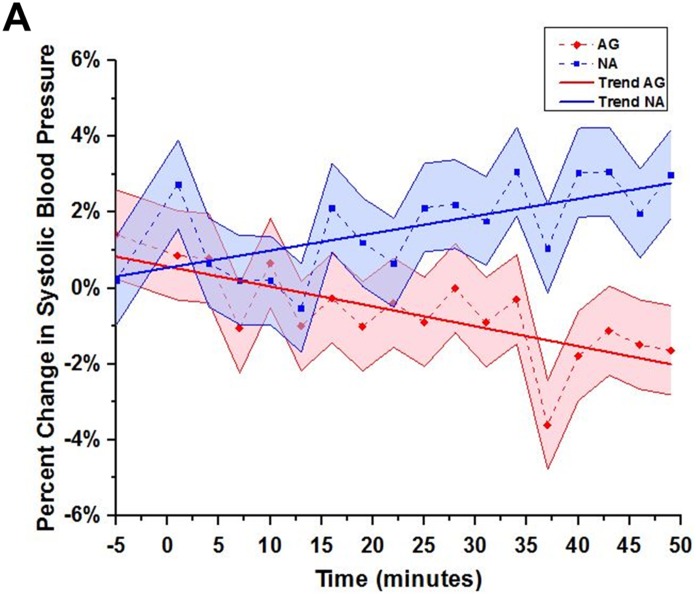
Systolic blood pressure response to violent media. Ag (red) individuals show reduction in systolic blood pressure while watching the violent media versus Na (blue) individuals who show progressive increase in systolic blood pressure. Systolic blood pressure measures were averaged for each group at each time point and a percent change and a trend line were calculated (Y-axis). Error bars (joined and filled) reflect the standard deviation of the data that are presented.

There were three 40-minute imaging conditions: resting baseline, where participants were instructed to rest with eyes open, a video presentation of violent scenes, and a video presentation of emotional scenes not portraying violence. The two videos (violent and emotional) were edited from R-rated movies and documentary films. The violent media presentation contained 20 scenes of violent acts encompassing the depiction of intentional acts of violence from one individual to another (e.g. interpersonal, shootings, street fights). The emotional media presentation contained 19 emotionally intense and action filled but non-violent scenes (e.g. people interacting during a natural disaster, sudden failures during competitive sports). The length of each of the violent or emotional scenes was between 1–4 minutes; these scenes were separated by a black screen that appeared for 30 seconds which signaled the next scene. The level of valence and intensity of each of the violent and emotional scenes was evaluated internally in the laboratory (data not shown) for valence and intensity and sequenced to optimize with the dynamics of FDG uptake (most intense scenes during the first 10 minutes of FDG uptake period). During the movie presentations, state levels of emotional reactivity were assessed using the Positive and Negative Affective Schedule (PANAS) with adjectives of mood states (ranked from 1, slightly to 5, extremely) [33]. The PANAS was completed by the subjects 5 minutes before the media presentations, 10 minutes into the presentations, and at the end of the media presentations. Table 2 shows PANAS adjectives where differences were found between the groups at p<0.05 during the violent as compared to emotional media presentations. Systolic and diastolic BP was monitored with a compression cuff that operated automatically (Propaq Encore) on the participant’s non-dominant arm starting 5 minutes before the imaging and continued throughout the scanning sessions occurring at 5-minute intervals. For Figure 1 systolic BP data was first averaged within each group at each point in the time series during the violent and during the emotional media presentation. Then, the percentage changes in BP (delta) were calculated from the emotional to the violent media within each group [(violent-emotional)/emotional].

**Table 2:** Statistical Parametric Mapping results showing the clusters where normalized brain metabolism was significantly different as a function of aggression.

Gyrus, Brodman Area (BA)	Talairach Coordinates(x, y, z)	Cluster size	Z-value^a^
**BASELINE (no media)**			
**Ag≥Na**			
Superior Temporal, BA 38	−36, 24, −36	210	4.79
Inferior Parietal, BA 40	−54, −46, 54	1960	5.75
Inferior Parietal, BA 40	42, −60, 44		5.16
Inferior Parietal	−32, −50, 50		5.43
Sensory Motor Area (SMA)	−8, −14, 64		4.13
Caudate	14, 26, −2	996	4.31
Posterior Cingulate, BA 30	−18, −58, 8		5.15
Precuneus	−14, −46, 44		5.03
Precuneus	4, −58, 50		4.73
Cuneus, BA 19	4, −76, 34	1500	5.46
Calcarine Gyrus	14, −76, 16		4.75
Superior Occipital Gyrus	−22, −72, 24		4.81
Cerebellum	−6, −88, −36		5.36
**Ag<Na**			
Orbitofrontal, BA 11	4, 50, −32	1849	5.27
Hippocampus	−18, 0, −38		4.98
Posterior Cerebellum	40, −66, −40	3795	4.31
Cerebellum V	14, −72, −36		4.13
**VIOLENT MEDIA**			
**Ag>Na**			
Superior Temporal, BA 38	52, 18, −28		4.68
Medial Temporal Pole	−36, 22, −36	613	5.11
Inferior Parietal, BA 40	−32, −36, 36	535	4.62
Fusiform Gyrus, BA 37	−34, −62, −8	2279	4.36
Superior Occipital Gyrus	−24, −76, 22		4.15
Lingual Gyrus	22, −54, 2		4.18
Caudate	14, 26, −2	436	4.38
**Ag<Na**			
Gyrus rectus, BA 11	2, 54, −30	924	5.20
Orbitofrontal, BA 11	22, 34, −26		3.91
Cerebellum	−8, −90, −36		6.12
**EMOTIONAL MEDIA**			
**Ag>Na**			
Lingual, BA 18	−20, −56, 4	2540	4.61

a Based on SPM8 cluster threshold of P<0.001, extent >100.

### PET Imaging

The 25 subjects were scanned 3 times with PET-FDG in counterbalanced order on separate days and under 3 conditions: resting baseline, violent scenes, non-violent emotional scenes. The scanning procedure is standardized and was described before [34]. The violent and neutral video presentations started 10 min prior to FDG injection and continued for a total of 40 min. PET imaging was conducted with a Siemens HR+ tomograph (resolution 4.5×4.5×4.5 mm^3^ full-width half-maximum, 63 slices) in 3D dynamic acquisition mode. Static emission scan started 35 min after FDG injection and continued for the next 20 min. Arterialized blood was used to measure FDG in plasma. During the uptake period of FDG, subjects were resting with eyes open (no stimulation) or watching a movie (violent or emotional) in a quiet dimly lit room with a nurse by their side to ensure that they did not fall asleep. Metabolic rates were computed using an extension of Sokoloff’s model [35]. The emission data for all the scans were corrected for attenuation and reconstructed using filtered back projection.

### Image and Data Analyses

**Figure 2:**
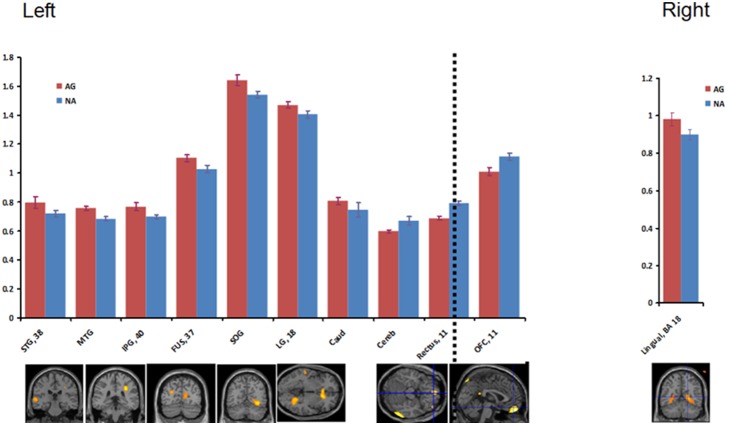
Glucose metabolism in response to media condition. Left panel: Relative glucose metabolism (Y-axis) in Ag (red) and Na (blue) in response to the violent media. On the left of the dotted line are results from Ag>Na contrast and on the right of the dotted line are results from the Ag<Na contrast. Right panel: Glucose metabolism results in response to the emotional media Ag>Na. There were no significant results for Ag<Na. Standard error is presented in the corresponding error bars.

Prior to the analysis, each participant’s PET image was mapped onto the Montreal Neurological Institute (MNI) template and smoothed via a Gaussian kernel with full width half maximum at 16 mm. Normalized metabolic images were analyzed using Statistical Parametric Mapping (SPM) [36]. The normalized images (relative images) were obtained by dividing the signal level of each voxel by the global mean, which was the average signal level of all voxels in the PET image. Analyses were performed in SPM8 with a flexible factor model design with one between-subject factor (Ag and Na groups) and one within-subject factor (baseline, violent, emotional conditions). Main effects of group were tested separately (Figure 2) as well as group x condition interactions. The cluster threshold used was p<0.001, cluster extent >100; given the number of subjects, these parameters were chosen to ensure a minimum of t = 3.00 for each cluster reported. After the SPM results were obtained, cubic regions of interest (ROIs) with 125 voxels were centered at the peak coordinates of relevant activation clusters to compute average metabolic values within these ROIs. Pearson linear correlations were used to assess the association between average ROI measures and BP.

The behavior and personality indices (Table 1) were analyzed using independent-samples t-tests Bonferroni corrected for multiple comparisons [37]. The changes in BP (delta) were calculated from the emotional to the violent media within each group [(violent-emotional)/emotional] (Figure 1). We tested whether the progressive change in systolic BP was significantly different between the groups with a general linear model (GLM), where time points and group were independent variables while the BP delta was the dependent variable. Two separate linear regression models were fitted within each group and used to test whether the delta in BP changed significantly over time and whether the slopes were significantly different between the groups. Analysis of PANAS responses to the violent and emotional media presentations was done by calculating differences in responses between violent and emotional presentations at 3 time points (pre, 10 min and end) using a GLM (Table 2).

## Results

### Traits, Inhibitory Control, and Resting Metabolism

As documented in Table 1, the groups were not different on demographics and media exposure and no differences were found on MPQ personality traits of PEM which includes the subscales *Well Being*, *Social Potency*, *Social Closeness* and *Achievement*. Not surprisingly, the groups were substantially different on *Negative Emotionality* and inhibitory control. Individuals from the Ag group, reported more NEM, with high scores on the NEM subscales, *Alienation*, *Aggression* and *Stress Reaction*. The Ag group also demonstrated poor inhibitory control, reporting less self-*Control* on the MPQ and also showed increased latency to respond specifically in the Conflict condition of the ANT. This performance measure of inhibitory control correlated with self-reported aggression such that more latency as a result of conflict in attention was seen in those with more trait aggression as measured by two different self-report scales (Buss-Perry *Physical Aggression* scale r = .76, P<0.0001, and MPQ *Aggression* (r = .66, P<0.001).

The normalized brain metabolic measures were characterized by robust group effects at resting baseline, involving hyperactivity in the DMN and caudate, and dampened OFC metabolism in Ag as compared to Na (Table 2). These resting metabolic measures in precuneus correlated positively across participants with NEM (R = .56, p<.01) and negatively with *Control* (R = −.46, 0<.05) whereas those in OFC showed the opposite pattern revealing a negative correlation with NEM (R = −.40, p<.05) and positive correlation with *Control* (R = .48, p<.05).

### Glucose Metabolism and Mood Reactivity during Media Viewing

Listed in Table 2 are the main effects of group for each condition separately. These results show similar group differences at resting baseline than for the comparisons during violent media presentation, involving hyperactivity in the DMN and caudate, and dampened OFC metabolism in Ag than Na participants (Figure 2, left panel). While viewing the emotional media presentation, the only significant difference between groups was higher glucose metabolism in bilateral lingual gyrus in the Ag group (Figure 2, right panel). Group x condition interactions were not significant at our threshold or at a reduced threshold of p<0.005.

**Figure 3:**
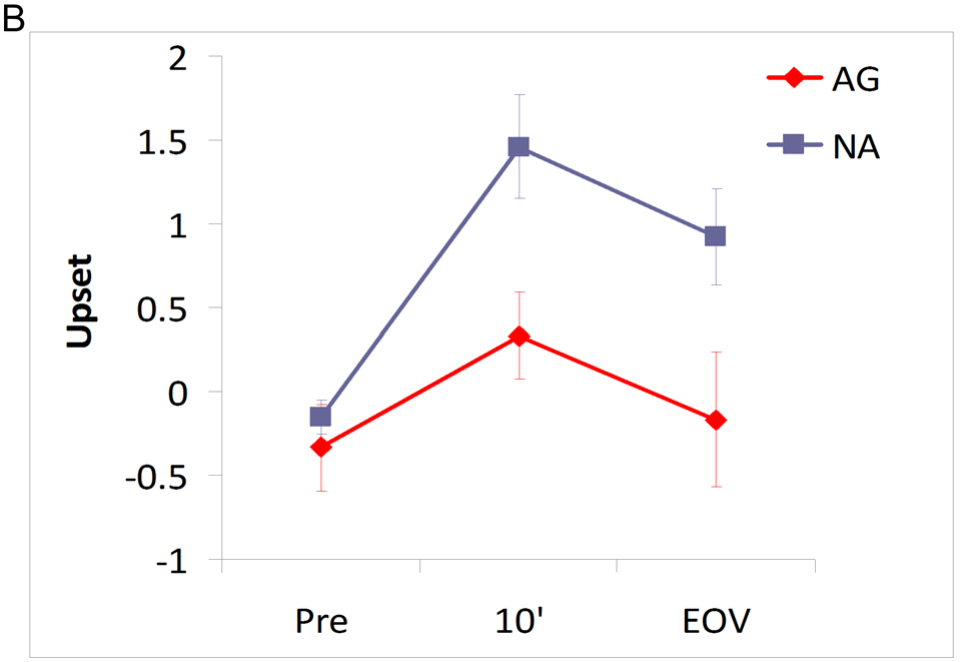
Time-course of emotional reactivity. Self-report of being *Upset* immediately before, during, and at the end (EOV) of the violent media viewing. Standard error is presented in the corresponding error bars.

As documented in Table 3, differences emerged between the groups in state reactivity 10 minutes into and at the end of the media presentations. During the violent media presentation as compared to the emotional media presentation, Ag participants when compared with the Na participants reported feeling less *Upset* (Figure 3) and *Nervous* and more *Inspired* and *Determined* (Table 3). In-line with the mood reactivity data, there were divergent responses between the groups in systolic BP across time. In the Na group, percent BP change progressively increased over time (t_16_ = 3.26, p = 0.002) while in the Ag group, systolic BP progressively decreased (t_16_ = −4.23, p = 0.0003) in response to the violent media as compared to emotional media (Figure 1). A comparison of the trend lines between the groups shows that the trend lines were significantly opposite (F_1, 32_ = 27.60, p<0.0001). Systolic and diastolic BP did not differ between the groups at resting baseline (p>0.05). Diastolic BP was not different between the groups in any of the conditions.

**Table 3:** Emotional reactivity during the violent media presentation.

PANAS^a^	Ag	Na	F and post hoc	
***Upset***				F_1,23_ = 6.58, P = 0.02
pre	1±0	1±0		
**10 min.**	**1.67±0.19**	**2.69±0.36**	**Ag<Na** **	
**End**	**1.33±0.19**	**2.08±0.26**	**Ag<Na** **	
***Nervous***				F_1,23_ = 3.64, P = 0.07
pre	1.58±0.29	1.54±0.24		
**10 min.**	**1.25±0.13**	**2.08±0.26**	**Ag<Na** **	
**End**	**1.33±0.14**	**2±0.25**	**Ag<Na** *	
***Inspired***				F_1,23_ = 4.64, P = 0.04
pre	2.58±0.34	2.31±0.31		
**10 minutes**	**2.25±0.39**	**1.31±0.17**	**Ag>Na** *	
End	2.33±0.45	1.61±0.24		
***Determined***				F_1,23_ = 7.56, P = 0.01
pre	3.42±0.40	2.62±0.33		
**10 min.**			**Ag>Na** **	
**End**	**3.08±0.47**	**1.77±0.28**	**Ag>Na** **	

a PANAS of response during violent media presentation using adjectives that demonstrated differences between the groups during the violent compared to emotional media; mean ± standard error.

*p<0.05,

**p<0.01.

**Figure 4:**
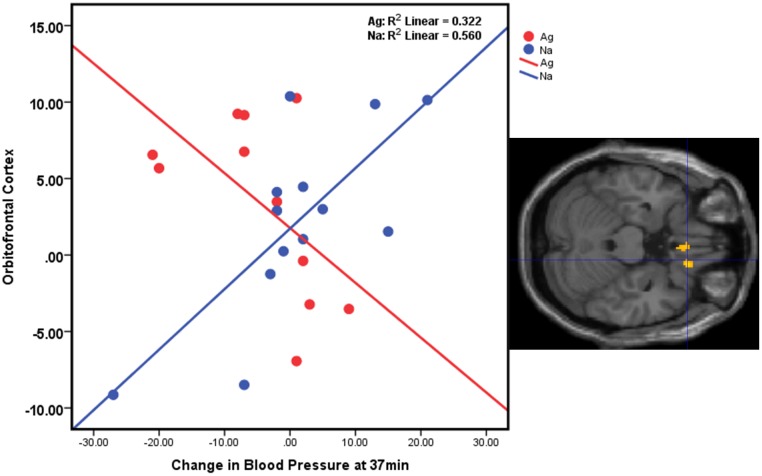
Coupling of blood pressure response with glucose metabolism in the OFC between the groups. On the y-axis is response in the OFC response to violent media compared with emotional media; on the x-axis is systolic BP change between violent media compared with emotional media at time 37 into the media viewing.

To examine the coupling of BP with glucose metabolism between the groups, we conducted ROI analyses to assess the correlation between regional metabolism during the violent media exposure and changes in systolic BP at time 37 (when most accentuated differences in BP were found between groups, as shown in Figure 1). In the Na, increases in BP were positively associated with increased metabolism in the right OFC (x = 22, y = 34, z = −26; r = 0.74; p<0.005) whereas the correlation was negative in (r = −0.56, p<0.005) (Figure 4) in whom decreases in BP were also associated with metabolism in precuneus (R = −.81, p<.001). That is, in Na participants increases in BP were associated with higher metabolism in OFC whereas in Ag participants decreases in BP were associated with increased metabolism in the OFC and precuneus.

## Discussion

This study documented brain, behavior, and blood-pressure response as a function of trait aggression. Results showed that Ag had heightened traits of NEM and poor inhibitory control compared to Na. These constitutional differences between the groups were apparent in their brain function at resting baseline and during the violent media viewing, where Ag had higher relative metabolism in the retrosplenial DMN, and lower relative metabolism in OFC, gyrus rectus, and posterior cerebellum. While watching the violent compared to emotional media, the Ag viewers reported being more *Inspired* and *Determined,* less *Upset* and *Nervous,* and showed a progressive decline in systolic blood-pressure compared with controls in whom systolic BP increased. Furthermore, the BP findings were differentially coupled with glucose metabolism between the groups. While viewing violent media, increased blood-pressure in Na was associated with increased metabolism in OFC; in Ag, the observed reduced blood-pressure was associated with increased metabolism in this same region and also in the precuneus.

### The Value of Pre-Selection Based on Abnormal Aggression Traits

In pre-selecting participants based on trait aggression this study revealed important baseline differences in brain and behavior compared with controls. Elevated trait aggression is found specifically in individuals with associated disorders, such as antisocial personality disorder and intermittent explosive disorder, as it has straightforward face validity [38]. In addition to elevated trait aggression, Ag also reported more *Alienation* and *Stress Reaction* and demonstrated poor inhibitory control, as measured by the ANT conflict [39], which are part of externalizing behaviors in adults [40]. Studies show that inhibitory control (as documented here using the ANT) play an important role in violent media effects and aggression [41]. Similarly, high levels of NEM as *Neuroticism* have shown robust connections with violence and aggression [30]. These results on characterizing personality in trait aggression, lend support to the GAM theory, documenting the specificity of trait aggression in its effects on other personality traits [42] and their potential cognitive substrates. Those who endorse few or no aggression items, hence, the Na group, scored at the norms in NEM and PEM, demonstrating that it is normative to endorse very few aggression questions, providing an adequate control for Ag. Importantly, PEM and its subscales were comparable between the groups, perhaps validating a characterization of trait aggression specifically involving NEM while having normative PEM [42]. Supportive of the GAM theory on the role of traits in media viewing, these trait results are important in setting the context of brain metabolism comparisons between the groups.

### Characterization of Trait Aggression through Resting Brain Metabolism

The most robust finding in this study is relative hyperactivity of the DMN during resting baseline with relative hypoactivity of the OFC and cerebellum in Ag compared to Na. The documented over-activity in components of the DMN may reflect a neural marker of enduring traits fostering inwardly directed attention to self-referential information stemming from years of social and cognitive learning [43]. Each of the DMN nodes and their network is associated with awareness and conscious information processing [44], mental imagery, perspective taking, and autobiographical memory retrieval [45]–[47] needed to facilitate an enduring brain activity pattern of behavioral patterns (i.e., trait) [48], [49]. Several studies mapped DMN regions with trait profiles; for example, *Neuroticism* (NEM in this study), was associated with lower volumetric measures and lower metabolism of the OFC [50], [51] in line with our results of hypoactive OFC in Ag. Conducting direct correlations between resting metabolism and NEM as well as with trait *Control*, we found that the lower resting metabolism in the OFC the higher were NEM and lower *Control* scores. In contrast the higher resting metabolism in precuneus the higher was NEM and lower *Control* trait scores. Supporting this finding are recent findings of higher precuneus with reduced conscientiousness and openness [49] both associated with NEM and characteristic of those with high trait aggression.

Other over activated regions at baseline among Ag participants included the sensory motor area and caudate. One could speculate that this increased activity during rest would have a role in compromised responses during a cognitive task. A recent study proposed that striatal dopamine circuits, particularly the caudate, may provide a mechanism for the active suppression of the DMN under conditions that require increased processing of external stimuli (e.g., an attention demanding cognitive task) relative to internal, self-directed processing [52]. This might be related to a recent finding where heightened trait aggression is associated with reduced dopamine in striatum [53] and that striatal dopamine influences the DMN to affect shifting between internal states and cognitive demands [54].

### Brain Metabolism during Violent Media Viewing

The fusiform gyrus was uniquely activated during violent media viewing in Ag, perhaps suggesting increased attention to facial representation of socially relevant cues [55]. Aside from the fusiform activation, while viewing the violent media presentation, the Ag participants compared with the Na showed similar patterns of activation as they had during resting baseline. As such, it appears that DMN regions are active during passive viewing of visual stimuli (e.g., movie) [56], [57]. We postulate that the violent media condition reflects congruence between the trait and the visual stimuli, such that the stimuli are syntonic (oscillating together) with internal processing, perhaps indicating personal experience with this material. Since resting baseline refers to mind wondering, it could be that participants in the Ag group have had aggressive thoughts that were instigating similar brain networks as during violent media viewing. A study in children during exposure to violent media documented engagement of the posterior cingulate and hippocampi, which was postulated to link memory and emotion to motor activation integrating existing aggression-related thoughts, thereby making them strongly accessible scripts over time [58]. The amygdala is a likely target for cortical arousal in violence viewing. Mathiak and Weber (2006) documented amygdala activation during active game-play in fMRI environment [59]. Their activation pattern showed signal decrease in the amygdala during players’ virtual violent behavior. Our study did not document amygdala responses possibly as a result of the passive nature of the viewing violent media or alternatively, amygdala was not documented because of the temporal resolution differences between PET and fMRI.

### Hypoactivity of the Orbitofrontal Cortex

In our study, the Ag participants showed a pattern of reduced OFC activity relative to the Na in the both resting baseline and violent media conditions. The OFC plays a role in externalizing/impulsive behavior, and regulating emotional and social behavior [13], [60]–[64]. Specific damage to the OFC is associated with impulsive and aggressive behavior [64], and individuals with such damage show little control over their emotions as well as limited awareness of the moral implications of their actions, and poor decision making [65]. Impulsive aggressive personality disordered patients demonstrate impaired emotion regulation, and exhibit blunted prefrontal, including OFC, metabolism in response to a serotonergic challenge [66]. Deficits in the orbitofrontal lobes as represented by atrophy, lesion, or hypoactive metabolism have been observed across a number of psychiatric populations prone to aggression (e.g., antisocial personality disorder, psychopathy, borderline personality disorder, intermittent explosive disorder) [66]–[68] and suggest that OFC hypo-function may be a common mechanism underlying the pathophysiology of aggressive behavior in general (e.g., both impulsive and premeditated forms). Hypoactivity of the OFC in this study and its correlation with high NEM and low *Control* scores further support the reliable implication of OFC in the externalizing continuum.

This OFC hypoactivity is consistent with other studies where exposure to violent media is associated with decreased OFC activation. In a study that examined components of the fronto-parietal network in response to aggressive video cues, reduced levels of OFC activation were found [19]. It is possible that OFC hypoactivation reflects desensitization to violence and disrupts the process of moral evaluation of the violent visual stimuli [69].

Familiarity with violent material could breed desensitization [69]–[71]. It could be that Ag have exhibited reduced inhibition and blunted evaluative categorization of violent stimuli as supported in other studies [71] such that they demonstrate a response (physiological/behavioral/cortical) that is suggestive of an overall desensitization to media violence [72], [73].

### Under-reactive Emotional and Autonomic Response to Violent Media

There is further evidence in this study supporting the desensitization hypothesis. The Ag group reported being less *Nervous* and *Upset* and more *Inspired* and *Determined* during the media violence (compared with emotional media) while their systolic BP progressively decreased. In stark contrast, The Na mood and BP responses to the violent media may be associated with a threat evaluation producing sympathetic activation, resulting in BP increase in the Na group. In a study with healthy adolescents, participants viewing violent movie clips experienced increased BP compared to baseline; however, prior exposure to violence was associated with lowered BP [21]. Autonomic under-arousal to threat stimuli has been documented in individuals who exhibit low levels of fear [74]. Angered subjects permitted to commit aggression against the person who had annoyed them often display a drop in systolic blood pressure. They seem to have experienced a physiological relaxation, as if they had satisfied their aggressive urges [75], [76].

Indeed, the documented pattern of BP under-reactivity in Ag was associated with hypoactivations in the OFC (Figure 3) and hyperactivation of the precuneus. Behaviorally-evoked changes in cardiovascular (e.g., blood pressure, heart rate) and cardiac-autonomic (e.g., heart rate variability) activity are correlated directly with neural activity within areas of the anterior cingulate cortex, OFC, medial prefrontal cortices, and the amygdala and often in interaction with activity in the insula, and relay regions of the thalamus and brainstem [22], [77], [78]. Based on neuroimaging and lesion evidence, a neurobiological model of cardiovascular reactivity shows that physiological and behavioral reactions are instantiated in the corticolimbic brains systems (e.g., medial/prefrontal corticies, insula, and amygdala) [79]. Afferent feedback, appraised by the OFC is integral in generation of somatic markers which trigger an emotional response, subsequently biasing overt behavior [80]. It is important to note here, that these results are relative to responses to emotional media viewing. It appears from our results that non-violent, yet emotionally salient action stimuli increase BP in the Ag individuals, whereas violent stimuli have the opposite effect of decreasing BP in these individuals. The specificity of hypo-response to violent content supports our assertion that the effects of violent media on individuals depend on theme-related traits, in this case aggression, and the brain of the beholder.

### Caveats

There are several limitations in this study that constrain our interpretation power and generalizability. First, there may have been too few participants in the study to ascertain group by condition interactions and to conduct correlations between trait and brain measures. Second, the inclusion of males only in this study was done to control for potentially differential emotional reaction patterns of activation as a function of sex. However, this approach prevents us from making any claims about female response to violent media. Future studies must include females. Third, the experimental design did not include an acute test of aggression following the media condition. Future studies could include such a test to document aggressive responses following violent media as a function of brain response during the violent media. Fourth, there are brain activity results during violent video games finding anterior cingulate involvement [59], [81]. These results may not be comparable to this study since playing video games requires task-dependent active attention compared to passive attention maintained during movie viewing as we show in our results; therefore more studies are needed to distinguish responses to media sources requiring active attention such as video games from those requiring only passive attention as movie scenes [82].

## References

[1] Bushman BJ, Geen RG (1990) Role of cognitive-emotional mediators and individual differences in the effects of media violence on aggression. J Pers Soc Psychol 58: 156–163. 2308072 10.1037//0022-3514.58.1.156

[2] Bushman BJ, Huesmann LR (2006) Short-term and long-term effects of violent media on aggression in children and adults. Arch Pediatr Adolesc Med 160: 348–352. 16585478 10.1001/archpedi.160.4.348

[3] Siever LJ (2008) Neurobiology of aggression and violence. Am J Psychiatry 165: 429–442. 18346997 10.1176/appi.ajp.2008.07111774 PMC4176893

[4] Shannon BJ, Raichle ME, Snyder AZ, Fair DA, Mills KL, et al (2011) Premotor functional connectivity predicts impulsivity in juvenile offenders. Proc Natl Acad Sci U S A 108: 11241–11245. 21709236 10.1073/pnas.1108241108 PMC3131347

[5] Raichle ME, MacLeod AM, Snyder AZ, Powers WJ, Gusnard DA, et al (2001) A default mode of brain function. Proc Natl Acad Sci U S A 98: 676–682. 11209064 10.1073/pnas.98.2.676 PMC14647

[6] Fox MD, Raichle ME (2007) Spontaneous fluctuations in brain activity observed with functional magnetic resonance imaging. Nat Rev Neurosci 8: 700–711. 17704812 10.1038/nrn2201

[7] Fox MD, Snyder AZ, Vincent JL, Raichle ME (2007) Intrinsic fluctuations within cortical systems account for intertrial variability in human behavior. Neuron 56: 171–184. 17920023 10.1016/j.neuron.2007.08.023

[8] Tomasi D, Volkow ND (2010) Functional connectivity density mapping. Proc Natl Acad Sci U S A 107: 9885–9890. 20457896 10.1073/pnas.1001414107 PMC2906909

[9] Berkowitz L (1984) Some effects of thoughts on anti- and prosocial influences of media events: a cognitive-neoassociation analysis. Psychol Bull 95: 410–427. 6399753

[10] Anderson CA, Bushman BJ (2002) Human aggression. Annu Rev Psychol 53: 27–51. 11752478 10.1146/annurev.psych.53.100901.135231

[11] Bartholow BD, Bushman BJ, Sestir MA (2006) Chronic violent video game exposure and desensitization to violence: Behavioral and event-related brain potential data. Journal of Experimental Social Psychology 42: 532–539.

[12] Anderson CA, Bushman BJ (2002) Psychology. The effects of media violence on society. Science 295: 2377–2379. 11923513 10.1126/science.1070765

[13] Bechara A, Damasio H, Damasio AR (2000) Emotion, decision making and the orbitofrontal cortex. Cereb Cortex 10: 295–307. 10731224 10.1093/cercor/10.3.295

[14] Christakou A, Brammer M, Giampietro V, Rubia K (2009) Right ventromedial and dorsolateral prefrontal cortices mediate adaptive decisions under ambiguity by integrating choice utility and outcome evaluation. J Neurosci 29: 11020–11028. 19726660 10.1523/JNEUROSCI.1279-09.2009 PMC6665528

[15] Spinella M (2002) Correlations among behavioral measures of orbitofrontal function. Int J Neurosci 112: 1359–1369. 12625195 10.1080/00207450290158250

[16] Bechara AB, Damasio H, Damasio AR, Lee GP (1999) Different contributions of the human amygdala and ventromedial prefrontal cortex to decision-making. Journal of Neuroscience 19: 5473–5481. 10377356 10.1523/JNEUROSCI.19-13-05473.1999 PMC6782338

[17] Yang Y, Raine A (2009) Prefrontal structural and functional brain imaging findings in antisocial, violent, and psychopathic individuals: a meta-analysis. Psychiatry Res 174: 81–88. 19833485 10.1016/j.pscychresns.2009.03.012 PMC2784035

[18] Goyer PF, Andreason PJ, Semple WE, Clayton AH, King AC, et al (1994) Positron-emission tomography and personality disorders. Neuropsychopharmacology 10: 21–28. 8179791 10.1038/npp.1994.3

[19] Strenziok M, Krueger F, Deshpande G, Lenroot RK, van der Meer E, et al (2011) Fronto-parietal regulation of media violence exposure in adolescents: a multi-method study. Soc Cogn Affect Neurosci 6: 537–547. 20934985 10.1093/scan/nsq079 PMC3190206

[20] Engelhardt CR, Bartholow BD, Saults JS (2011) Violent and nonviolent video games differentially affect physical aggression for individuals high vs. low in dispositional anger. Aggress Behav 37: 539–546. 21905039 10.1002/ab.20411

[21] Madan A, Mrug S, Wright RA (2014) The effects of media violence on anxiety in late adolescence. J Youth Adolesc 43: 116–126. 24014349 10.1007/s10964-013-0017-3

[22] Gianaros PJ, Sheu LK, Remo AM, Christie IC, Crtichley HD, et al (2009) Heightened resting neural activity predicts exaggerated stressor-evoked blood pressure reactivity. Hypertension 53: 819–825. 19273741 10.1161/HYPERTENSIONAHA.108.126227 PMC2674279

[23] Buss AH, Perry M (1992) The aggression questionnaire. J Pers Soc Psychol 63: 452–459. 1403624 10.1037//0022-3514.63.3.452

[24] Oldfield RC (1971) The assessment and analysis of handedness: the Edinburgh inventory. Neuropsychologia 9: 97–113. 5146491 10.1016/0028-3932(71)90067-4

[25] Hollingshead AB (1975) Four-factor index of social status. Unpublished paper.

[26] Wilkinson G (1993) The Wide-Range Achievement Test 3: Administration Manual. Wilminton, DE: Wide-Range Inc.

[27] Wechsler D (1999) Wechsler abbreviated scale of intelligence: San Antonio, TX: Psychological Corporation.

[28] Beck AT, Steer RA, Brown GK. (1996) Beck Depression Inventory Manual. 2nd ed. San Antonio, TX: The Psychological Corporation.

[29] Tellegen A, Waller NG (1997) Exploring personality through test construction: development of the multidimensional personality questionnaire. In: Briggs SR, Cheek JM, editors. Personality measures: development and evaluation. Greenwich: JAI Press.

[30] Blonigen DM, Krueger RF. (2007) Personaity & Violence: The unifying role of structural models of personality. In: Flannery DJ VA, Waldman ID (Eds.), editor. The Cambridge handbook of violent behavior. New York, NY: Cambridge University Press.

[31] Hicks BM, Markon KE, Patrick CJ, Krueger RF, Newman JP (2004) Identifying psychopathy subtypes on the basis of personality structure. Psychol Assess 16: 276–288. 15456383 10.1037/1040-3590.16.3.276

[32] Fan J, McCandliss BD, Sommer T, Raz A, Posner MI (2002) Testing the efficiency and independence of attentional networks. Journal of Cognitive Neuroscience 14: 340–347. 11970796 10.1162/089892902317361886

[33] Watson D, Clark LA, Tellegen A (1988) Development and validation of brief measures of positive and negative affect: the PANAS scales. J Pers Soc Psychol 54: 1063–1070. 3397865 10.1037//0022-3514.54.6.1063

[34] Wang GJ, Volkow ND, Roque CT, Cestaro VL, Hitzemann RJ, et al (1993) Functional importance of ventricular enlargement and cortical atrophy in healthy subjects and alcoholics as assessed with PET, MR imaging, and neuropsychologic testing. Radiology 186: 59–65. 8416587 10.1148/radiology.186.1.8416587

[35] Phelps ME, Hoffman EJ, Coleman RE, Welch MJ, Raichle ME, et al (1976) Tomographic images of blood pool and perfusion in brain and heart. J Nucl Med 17: 603–612. 818345

[36] Friston KJ, Holmes AP, Worsley KJ, Poline JB, Frith CD, et al (1995) Statistical parametric maps in functional imaging: a general approach. Human Brain Mapping 2: 189–210.

[37] Stevens J (1992) Applied multivariate statistics for the social sciences. 2nd ed. Lawrence Erlbaum Associates: NewJersey.

[38] Anderson CA, Buckley KE, Carnagey NL (2008) Creating your own hostile environment: a laboratory examination of trait aggressiveness and the violence escalation cycle. Pers Soc Psychol Bull 34: 462–473. 18340032 10.1177/0146167207311282

[39] Thienel R, Voss B, Kellermann T, Reske M, Halfter S, et al (2009) Nicotinic antagonist effects on functional attention networks. Int J Neuropsychopharmacol 12: 1295–1305. 19737441 10.1017/S1461145709990551

[40] Krueger RF, Markon KE, Patrick CJ, Benning SD, Kramer MD (2007) Linking antisocial behavior, substance use, and personality: an integrative quantitative model of the adult externalizing spectrum. J Abnorm Psychol 116: 645–666. 18020714 10.1037/0021-843X.116.4.645 PMC2242625

[41] Swing EL, Anderson CA (2014) The role of attention problems and impulsiveness in media violence effects on aggression. Aggress Behav. 10.1002/ab.21519 24452487

[42] Hosie J, Gilbert F, Simpson K, Daffern M (2014) An examination of the relationship between personality and aggression using the general aggression and five factor models. Aggress Behav 40: 189–196. 24497001 10.1002/ab.21510

[43] Nagai Y, Critchley HD, Featherstone E, Trimble MR, Dolan RJ (2004) Activity in ventromedial prefrontal cortex covaries with sympathetic skin conductance level: a physiological account of a “default mode” of brain function. Neuroimage 22: 243–251. 15110014 10.1016/j.neuroimage.2004.01.019

[44] Zhang S, Li CS (2012) Functional connectivity mapping of the human precuneus by resting state fMRI. Neuroimage 59: 3548–3562. 22116037 10.1016/j.neuroimage.2011.11.023 PMC3288461

[45] Raichle ME, Snyder AZ (2007) A default mode of brain function: a brief history of an evolving idea. Neuroimage 37: 1083–1090 discussion 1097–1089. 17719799 10.1016/j.neuroimage.2007.02.041

[46] Dorfel D, Werner A, Schaefer M, von Kummer R, Karl A (2009) Distinct brain networks in recognition memory share a defined region in the precuneus. Eur J Neurosci 30: 1947–1959. 19895564 10.1111/j.1460-9568.2009.06973.x

[47] McDermott KB, Ojemann JG, Petersen SE, Ollinger JM, Snyder AZ, et al (1999) Direct comparison of episodic encoding and retrieval of words: an event-related fMRI study. Memory 7: 661–678. 10659091 10.1080/096582199387797

[48] Wei L, Duan X, Zheng C, Wang S, Gao Q, et al (2014) Specific frequency bands of amplitude low-frequency oscillation encodes personality. Hum Brain Mapp 35: 331–339. 22987723 10.1002/hbm.22176 PMC6869309

[49] Sampaio A, Soares JM, Coutinho J, Sousa N, Goncalves OF (2013) The Big Five default brain: functional evidence. Brain Struct Funct. 10.1007/s00429-013-0610-y 23881294

[50] Wright CI, Williams D, Feczko E, Barrett LF, Dickerson BC, et al (2006) Neuroanatomical correlates of extraversion and neuroticism. Cereb Cortex 16: 1809–1819. 16421327 10.1093/cercor/bhj118

[51] DeYoung CG, Hirsh JB, Shane MS, Papademetris X, Rajeevan N, et al (2010) Testing predictions from personality neuroscience. Brain structure and the big five. Psychol Sci 21: 820–828. 20435951 10.1177/0956797610370159 PMC3049165

[52] Kelly C, deZubicaray G, Di Martino A, Copland DA, Reiss PT, et al (2009) L-dopa modulates functional connectivity in striatal cognitive and motor networks: a double-blind placebo-controlled study. J Neruosci 29: 7364–7378. 10.1523/JNEUROSCI.0810-09.2009 PMC2928147 19494158

[53] Schluter T, Winz O, Henkel K, Prinz S, Rademacher L, et al (2013) The impact of dopamine on aggression: an [^18^F]-FDOPA PET Study in healthy males. J Neurosci 33: 16889–16896. 24155295 10.1523/JNEUROSCI.1398-13.2013 PMC6618436

[54] Dang LC, Donde A, Madison C, O’Neil JP, Jagust WJ (2012) Striatal dopamine influences the default mode network to affect shifting between object features. J Cogn Neurosci 24: 1960–1970. 22640392 10.1162/jocn_a_00252 PMC3510672

[55] Wiggett AJ, Downing PE (2011) Representation of action in occipito-temporal cortex. J Cogn Neurosci 23: 1765–1780. 20807060 10.1162/jocn.2010.21552

[56] Shulman GL, Fiez JA, Corbetta M, Buckner RL, Miezin FM, et al (1997) Common Blood Flow Changes across Visual Tasks: II. Decreases in Cerebral Cortex. J Cogn Neurosci 9: 648–663. 23965122 10.1162/jocn.1997.9.5.648

[57] Mazoyer B, Zago L, Mellet E, Bricogne S, Etard O, et al (2001) Cortical networks for working memory and executive functions sustain the conscious resting state in man. Brain Research Bulletin 54: 287–298. 11287133 10.1016/s0361-9230(00)00437-8

[58] Murray JP, Liotti M, Ingmundson PT, Mayberg HS, Pu Y, Zamarripu F, et al (2006) Children’s brain activations while viewing televised violence revealed by fMRI. Media Psychology 8: 25–37.

[59] Mathiak K, Weber R (2006) Toward brain correlates of natural behavior: fMRI during violent video games. Hum Brain Mapp 27: 948–956. 16628606 10.1002/hbm.20234 PMC6871426

[60] Bechara A, Damasio AR, Damasio H, Anderson SW (1994) Insensitivity to future consequences following damage to human prefrontal cortex. Cognition 50: 7–15. 8039375 10.1016/0010-0277(94)90018-3

[61] Bechara A, Damasio H, Tranel D, Damasio AR (1997) Deciding advantageously before knowing the advantageous strategy. Science 275: 1293–1295. 9036851 10.1126/science.275.5304.1293

[62] Rudebeck PH, Bannerman DM, Rushworth MF (2008) The contribution of distinct subregions of the ventromedial frontal cortex to emotion, social behavior, and decision making. Cogn Affect Behav Neurosci 8: 485–497. 19033243 10.3758/CABN.8.4.485

[63] Davidson RJ, Putnam KM, Larson CL (2000) Dysfunction in the neural circuitry of emotion regulation-A possible prelude to violence. Science 289: 591–572. 10915615 10.1126/science.289.5479.591

[64] Izquierdo A, Suda RK, Murray EA (2005) Comparison of the effects of bilateral orbital prefrontal cortex lesions and amygdala lesions on emotional responses in rhesus monkeys. J Neurosci 25: 8534–8542. 16162935 10.1523/JNEUROSCI.1232-05.2005 PMC6725674

[65] Bechara A (2004) The role of emotion in decision-making: evidence from neurological patients with orbitofrontal damage. Brain Cogn 55: 30–40. 15134841 10.1016/j.bandc.2003.04.001

[66] New AS, Hazlett EA, Buchsbaum MS, Goodman M, Reynolds D, et al (2002) Blunted prefrontal cortical fluorodeoxyglucose Positron Emission Tomography Response to Meta-Chlorophenylpiperazine in impulsive aggression. Archives of General Psychiatry 59: 621–629. 12090815 10.1001/archpsyc.59.7.621

[67] Raine A, Meloy RJ, Bihrle S, Stoddard J, LaCasse L, et al (1998) Reduced prefrontal and increased subcortical brain functioning assessed using positron emission tomography in predatory and affective murderers. Behavioral Sciences and the Law 16: 319–332. 9768464 10.1002/(sici)1099-0798(199822)16:3<319::aid-bsl311>3.0.co;2-g

[68] Siever LJ, Buchsbaum MS, New AS, Spiegel-Cohen J, Wei T, et al (1999) d,l-fenfluramine Response in Impulsive Personality Disorder Assessed with [^18^F]fluorodeoxyglucose Positron Emission Tomography. Neuropsychopharmacology 20: 413–423. 10192822 10.1016/S0893-133X(98)00111-0

[69] Funk JB (2005) Children’s exposure to violent video games and desensitization to violence. Child Adolesc Psychiatr Clin N Am 14: 387–404, vii–viii. 10.1016/j.chc.2005.02.009 15936665

[70] Huesmann LR, Kriwil L (2007) Why observing violence increases the risk of violent behavior in the observer; (Ed.) DF, editor. Cambridge, UK: Cambridge University Press.

[71] Fanti KA, Vanman E, Henrich CC, Avraamides MN (2009) Desensitization to media violence over a short period of time. Aggress Behav 35: 179–187. 19172659 10.1002/ab.20295

[72] Bailey K, West R, Anderson CA (2011) The association between chronic exposure to video game violence and affective picture processing: an ERP study. Cogn Affect Behav Neurosci 11: 259–276. 21461985 10.3758/s13415-011-0029-y

[73] Kelly CR, Grinband J, Hirsch J (2007) Repeated exposure to media violence is associated with diminished response in an inhibitory frontolimbic network. PLoS One 2: e1268. 18060062 10.1371/journal.pone.0001268 PMC2092389

[74] Raine A (1996) Autonomic nervous system factors underlying disinhibited antisocial and violent behavior. Annals New York Academy of Sciences: 46–60. 10.1111/j.1749-6632.1996.tb32508.x 8853591

[75] Hokanson JE, Burgess M (1962) The effects of status, type of frustration, and aggression on vascular processes. J Abnorm Soc Psychol 65: 232–237. 13961249 10.1037/h0043800

[76] Hokanson JE, Burgess M (1962) The effects of three types of aggression on vascular processes. J Abnorm Soc Psychol 64: 446–449. 13908281 10.1037/h0040236

[77] Critchley HD (2005) Neural mechanisms of autonomic, affective, and cognitive integration. J Comp Neurol 493: 154–166. 16254997 10.1002/cne.20749

[78] Mujica-Parodi LR, Korgaonkar M, Ravindranath B, Greenberg T, Tomasi D, et al (2009) Limbic dysregulation is associated with lowered heart rate variability and increased trait anxiety in healthy adults. Hum Brain Mapp 30: 47–58. 18041716 10.1002/hbm.20483 PMC2993012

[79] Gianaros PJ, Sheu L (2009) A review of neuroimaging studies of stressor-evoked blood pressure reactivity: Emerging evidence for a brain-body pathway to coronary heart disease risk. NeuoImage 47. 10.1016/j.neuroimage.2009.04.073 PMC2743251 19410652

[80] Damasio A (1994) Descartes’ Error: Emotion, Reason, and the Human Brain. New York: HarperCollins.

[81] Chou YH, Yang BH, Hsu JW, Wang SJ, Lin CL, et al (2013) Effects of video game playing on cerebral blood flow in young adults: a SPECT study. Psychiatry Res 212: 65–72. 23137807 10.1016/j.pscychresns.2012.10.002

[82] Carnagey NL, Anderson CA (2007) Changes in attitudes towards war and violence after September 11, 2001. Aggress Behav 33: 118–129. 17441012 10.1002/ab.20173

